# The adaptive significance of population differentiation in offspring size of the least killifish, *Heterandria formosa*

**DOI:** 10.1002/ece3.509

**Published:** 2013-03-05

**Authors:** Jeff Leips, F Helen Rodd, Joseph Travis

**Affiliations:** 1 Department of Biological Sciences, University of Maryland Baltimore County1000 Hilltop Circle, Baltimore, MD, 21250, USA; 2 Department of Biological Science, Florida State UniversityTallahassee, Florida, 32306, USA; 3 Department of Ecology and Evolutionary Biology, University of TorontoToronto, M5S 3G5, Canada

**Keywords:** Behavior, competition, competitive ability, density dependence, growth rates, life history evolution, life-history traits, maternal effects

## Abstract

We tested the hypothesis that density-dependent competition influences the evolution of offspring size. We studied two populations of the least killifish (*Heterandria formosa*) that differ dramatically in population density; these populations are genetically differentiated for offspring size, and females from both populations produce larger offspring when they experience higher social densities. To look at the influences of population of origin and relative body size on competitive ability, we held females from the high-density population at two different densities to create large and small offspring with the same genetic background. We measured the competitive ability of those offspring in mesocosms that contained either pure or mixed population treatments at either high or low density. High density increased competition, which was most evident in greatly reduced individual growth rates. Larger offspring from the high-density population significantly delayed the onset of maturity of fish from the low-density population. From our results, we infer that competitive conditions in nature have contributed to the evolution of genetically based interpopulation differences in offspring size as well as plasticity in offspring size in response to conspecific density.

## Introduction

Propagule size is a key life-history trait that directly influences both offspring and maternal fitness. Under many conditions, larger offspring have higher individual fitness (Bagenal 1969; Tessier and Consolati 1989; Fox and Czesak 2000; Janzen and Warner 2009; Marshall and Keough 2009; Jacobs and Sherrard 2010; Rius et al. 2010), but there are some circumstances in which the benefit of larger size is either very small or undetectable (e.g., Kaplan 1992; Fox et al. 1997; Bashey 2006). As offspring size is often inversely related to offspring number, variation in offspring size will also create variation in parental fitness through the tradeoff between offspring size and number. High variation in offspring size among conspecific populations and related species (Reznick and Endler 1982; Amat et al. 2001; Marshall and Keough 2007; Reed et al. 2009; Riesch et al. 2009; Collin and Salazar 2010) raises the questions of how much of this variation is directly adaptive, under which circumstances might it be adaptive, and for whom, parent or offspring, does the bulk of any benefit accrue.

Evolutionary theories that consider offspring size explore conditions under which females should partition resources between offspring size and number to maximize parental fitness (Smith and Fretwell 1974; Brockelman 1975; Lloyd 1987; Winkler and Wallin 1987; Kindsvater et al. 2010). Theory predicts that the optimal parental investment per offspring should change when the relationship between offspring size and offspring fitness differs across ecological settings (Parker and Begon 1986; Sibly and Calow 1986; McGinley et al. 1987). Population density is one ecological factor that theory predicts will play an important role in selecting for variation in offspring size and number (Brockelman 1975; Parker and Begon 1986). In high-density populations, when competition is intense, selection favors the production of larger, competitively superior offspring (reviewed in Stearns 1976), especially when the effects of density affect juveniles more than adults (Sibley and Calow 1983). This prediction assumes that larger body size at offspring independence confers higher fitness at higher densities.

Empirical evidence supports the idea that population density can affect the relationship between offspring size and fitness. In plants, large seeds and seedlings have higher fitness in high-density environments (e.g., Harper 1977; Stanton 1984; Winn and Miller 1995). Fewer studies have examined this relationship in animals, but their results uphold this general pattern (Berven and Chadra 1988; Perrin 1989; Hutchings 1991; Einum and Fleming 1999; Marshall et al. 2006; Bashey 2008). Maternal adjustments of offspring size in response to conspecific density (or indicators of density) also suggest that density is an important agent of selection on offspring size. For example, when food is limited, females often produce fewer but larger offspring (Gliwicz and Guisande 1992; Reznick and Yang 1993; Reznick et al. 1996; Bashey 2006). Studies that manipulate adult densities (Kawecki 1995; Allen et al. 2008; Leips et al. 2009) and chemicals associated with variation in density (Burns 1995) find that larger offspring are produced when females experience high-density conditions. An adaptive interpretation of this pattern is that the larger offspring are better competitors in the higher densities that they will face. Despite the attention given to offspring size variation and the adaptive significance of this variation, few studies test both facets of this issue (but see Reznick 1982; Reznick et al. 1990, 2001; Bashey 2006).

Here, we examine the relationships among offspring size, density, and components of offspring fitness using the least killifish (*H. formosa*) from two natural populations in north Florida, Trout Pond (TP) and the Wacissa River (WR). These populations differ dramatically in conspecific density (Leips and Travis 1999; Richardson et al. 2006), with population density in the Wacissa River often exceeding that of Trout Pond by as much as sevenfold during the breeding season. Fish from these populations also produce offspring that differ substantially in size; wild caught Wacissa River females have fewer but larger (>40%) offspring per clutch (Leips and Travis 1999; Schrader and Travis 2005) and these differences have a genetic basis (Leips et al. 2000). However, size-specific allocation of mass devoted to reproduction, after adjusting for differences in female body size, does not differ between populations. Thus, the differences in the numbers and sizes of offspring produced reflect evolved differences in the way that reproductive mass is packaged, suggesting that these traits have responded to population-specific selection pressures.

Our study addresses three general questions related to the adaptive significance of offspring size variation in different competitive environments. First, do offspring from high-density populations perform better (exhibit higher growth rates, mature earlier, and/or mature at larger body size) in high-density conditions than offspring from low-density populations? Second, do offspring from populations that differ in density differ in their density-dependent competitive ability when placed in competition with each other? Third, how important is the relative difference in body size in determining the outcome of competition? When females from both of these populations experience high adult density, they produce larger offspring than females held at a lower adult density (Leips et al. 2009). We took advantage of this plastic response of offspring size to social density to manipulate the size of offspring from the Wacissa River population. This allowed us to examine the role of relative size versus population of origin in determining the outcome of density-dependent competition. It also allowed us to evaluate the extent to which maternal adjustments of offspring size is an adaptive response to variation in competitive environments.

## Methods

### Study organism and populations

**Figure 1:**
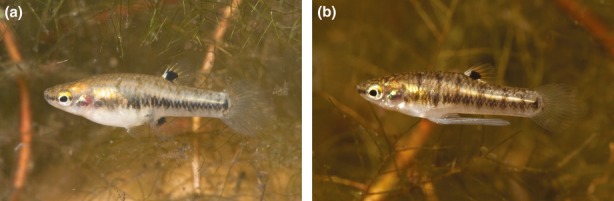
(A) Adult female *Heterandria formosa*. (B) Adult male *H. formosa*.

*Heterandria formosa* is a live-bearing topminnow in the Poeciliid family that tends to aggregate in aquatic vegetation in shallow water. Their small size and external morphological features indicating sexual maturity make *H. formosa* an excellent species for life-history studies. In females (Fig. 1A), sexual maturity is visually indicated by the appearance of a small black dot on the anal fin (Fraser and Renton 1940); they mature between 8 and 10 mm (standard length) in approximately 30–40 days. In males (Fig. 1B), sexual maturity is indicated by the development of the gonopodium, a modified anal fin used for sperm transfer (Constanz 1989); they take longer to mature (40–50 days) and mature at a larger body size (10–14 mm) than females.

Parental fish for this experiment were obtained from the Wacissa River (WR) in Jefferson County and Trout Pond (TP) in Leon County. These populations are approximately 50 km apart and genetically isolated (Baer 1998; Soucy and Travis 2003), although molecular data suggest that this isolation is fairly recent (Baer 1998).

### Animal husbandry

We measured offspring performance traits using second generation (F_2_), lab-reared offspring. Parental stocks of WR and TP fish were initiated with 50 wild caught females and 20 wild caught males from each population. Breeding was carried out in 19L aquaria in a temperature (31°C) and light (14:10 day:night) controlled laboratory. Parental densities were maintained at 5–6 females and 2–3 males per tank during breeding of the parental generation, and tanks were checked weekly for offspring. First generation (F_1_) offspring were removed and assigned to an F_1_ density treatment (explained below). Pedigrees were kept for all offspring so that inbreeding in the F_1_s could be minimized. Only individuals that were born in different tanks were allowed to mate.

Females in the WR F1 breeding tanks were kept at one of two densities in 19L aquaria (either 6 females and 2 males per tank, or 2 females and 2 males per tank). These different maternal densities are within the range of natural densities experienced by WR fish (Leips and Travis 1999) and influence offspring size of both WR and TP females (Leips et al. 2009). All TP F1 breeding tanks had 6 females and 2 males, thus producing large TP offspring. Fish were fed ground Tetramin® (Tetra, Melle, Germany) flake food twice daily ad libitum, so any effects on offspring phenotype from different female densities should have resulted from density differences alone, not from reduced per capita resources. To summarize, three types of F_2_ offspring were produced: TP offspring from tanks with high female density, WR offspring from tanks with high (WR[H]) density, and WR offspring from tanks with low (WR[L]) density.

### Experimental design

Eight treatments were used in a two factor, fractional factorial design (Dunson and Travis 1991; Warner et al. 1991) (Table S1). Treatments evaluated offspring performance (growth rate, age, and body size at maturity) at either low or high density (8 and 32 individuals) in treatments containing either offspring from a single population (TP, or WR(H)) or offspring from both populations in competition (WR[H] with TP, or WR[L] with TP). Thus, there were four single population treatments and four mixed population treatments. Single population treatments evaluated performance of the TP and WR(H) offspring at low and high densities. Two of the mixed population treatments measured the performance of the larger WR(H) offspring when in competition against the smaller TP offspring at both densities. The other two mixed population treatments compared the performance of WR(L) offspring when in competition with TP offspring at both densities. In this case, the offspring were more similar in body size. In each mixed population treatment, TP and WR offspring were mixed 1:1 such that there were either 8 or 32 individuals per unit, matching the total density of the single population treatments.

### Execution of the competition experiment

Prior to the initiation of a set of experimental replicates, all F_2_ offspring were removed from F_1_ breeding tanks. We then collected offspring for assignment to the different treatment groups for the next 8 days. Thus, the offspring within each treatment were similar, but not identical, in age. A drawback of this procedure is that it minimized our ability to detect age-specific trait differences among treatments because trait measurements were taken weekly. However, our results should not be biased because all treatments used offspring within this range of ages. A subset of offspring from each treatment was photographed to determine initial body size. In the mixed population treatments, we tagged fish from one of the populations with calcein to identify the origin of the fish in subsequent sampling events. WR fish were marked in three of the replicates, TP fish in the remaining two. Calcein binds to calcium in tissues and can be seen in fin rays and scales (Leips et al. 2001). *H. formosa* were tagged by maintaining individuals for 24 h in a solution of 250 mg/L solution of calcein, buffered to a pH of 5.3 using tris (hydroxymethyl) amino-methane. This dye is retained for at least 5 weeks in the laboratory and had no adverse effects on survival or growth compared to an equal number of unmarked control fish (Leips et al. 2001).

One set of the 8 treatments was started in aquaria in the laboratory at one time, and was then moved to the experimental mesh bags placed in large (850L) cattle watering tanks in an open field at the Florida State University Greenhouse Facility. Each tank contained a mixture of well water and rainwater as well as aquatic vegetation and plankton from Trout Pond. Tank communities were established in five cattle tanks 8 months prior to the initiation of the experiment. Approximately, 2000 mL of suspended plankton from Trout Pond were added to each tank monthly during the course of the experiment.

Each cattle tank contained a set of 8 mesh bags in which the fish from one replicate of each of the 8 treatments were raised. Bags were made from nylon tulle material (1.5 mm mesh size) and framed with glass rods to maintain a rectangular shape. Bags were hung from cords attached to a square PVC frame, and suspended in the tank so that each contained approximately 16L of water (Fig S1). Each bag contained a small amount of submerged vegetation, *M. laxum* and *H. verticillata*, to provide cover and substrate for epiphytic growth. *H. formosa* is omnivorous, and fish in the bags presumably fed on plankton as well as the vegetation and algae in the bags.

Each treatment was replicated 5 times (one replicate per cattle tank). Each time a set of replicates was started, all 8 treatments were initiated in separate 19L aquaria (in 16L of water) in the laboratory at the same time. For the first week, fish in the low-density treatments (cells 1,3,4, and 7 in Table 2A1, Table S1) were fed twice daily with 10 mg of flake food (an ad libitum amount of food). Fish were fed 15 mg twice daily for the second week, and then 20 mg if individuals were retained in the lab for a third week (see rationale below). High-density treatments (cells 2, 5, 6, and 8 in Table 2A1, Table S1) received the same ration per aquarium (i.e., one quarter of the food ration per individual). This confounds differences in per capita food amounts and density, but was meant to reflect the effect of increasing density in natural populations. Thus, both resource availability and differences in social environment may have influenced the measured traits.

Fish were moved to a cattle tank when they had grown too large to escape through the mesh bags (2–3 weeks). Each replicate was then censused every week. This sampling interval reduces our power to detect subtle differences among the treatments but was necessary for logistical reasons. Males, females, and immature individuals were sorted by visual inspection and taken to the lab. Calcein tagged and untagged fish were sorted by examining anesthetized individuals under an epifluorescence microscope (Leips et al. 2001). Fish were anesthetized in a low dose of MS-222. In the third sampling period, all calcein-tagged fish were retagged with calcein to ensure that the tag was retained until the next sampling period. Fish in the different categories (tagged and untagged; mature male, mature female, and immature individuals) from all treatments were photographed separately in water-filled bins containing a metric scale and then returned to the cattle tanks. The size of fish in each group was measured from photographs using an image analysis system.

The entire experiment was carried out over the course of a single summer The monitoring period of each replicate ran from the date the replicate was set up until the first female in each bag had reached sexual maturity (or the first TP and WR females had matured in the mixed treatment bags).

### Statistical analysis

The initial sizes of the offspring from all three treatments (TP: *n* = 29; WR[H]: *n* = 19, and WR[L]: *n* = 10) were compared using analysis of variance, with population as the categorical variable (Zar 2009). Tukey's HSD procedure was used for post hoc comparisons of means among the groups. We measured the initial size of fish only in the first replicate to minimize handling of newborn offspring. As the effect of maternal density on offspring size matched that of our previous experiments (Leips et al. 2009), we assumed the maternal density treatment had similar effects on offspring size in the remaining replicates.

We assayed the effects of the different treatments on offspring performance using three traits: the average growth rates of individuals in each group for the first 2 weeks, and the age and size of the first maturing individual of each group from a replicate. Growth rates were calculated only for the first 2 weeks because growth rates of males and females begin to diverge after that time (Parrino, Leips and Travis, unpubl. ms.). Focusing only on growth rate for the first 2 weeks minimizes the effects of chance differences in sex ratio among replicates that could have obscured differences among treatment groups. Average trait values of maturing individuals from each replicate were not used for analyses of age and size at maturity because there was large variation in the proportion of individuals that matured in each replicate. This confounded age-structure with density effects among replicates. In addition, in some cases (particularly in high-density treatments), maturation occurred over a long period of time, skewing the mean and median values differently in the different replicates.

We carried out four analyses of variance for each trait using contrast codes to explore the role of maternal density, offspring density, and population of origin on offspring traits. All analyses (designated as A–D) and associated contrasts are detailed in Table S1. Within any single analysis (A, B, C, or D), there are three contrasts i, ii, and iii. The first two contrasts (i and ii) test for main effects and the third contrast (iii) tests for the interaction between two main effects.

Analysis A uses cells 1, 2, 4, and 6 (Table 2A1, Table S1): the treatments with offspring from only one stock population (either TP or WR(H)), at two densities, low (*N* = 8 individuals) or high (4*N* = 32 individuals). For any dependent variable (growth rate, age, or size at maturity), contrast i tests the effect of quadrupling the density. Contrast ii tests whether TP offspring differ from WR(H). Contrast iii tests whether the effect of quadrupling density differs between stocks, thus testing if the populations are differentially sensitive to the depressant effect of density.

Analysis B uses cells 1, 2, 3, and 5 and only uses data from TP offspring. Contrast i tests whether TP traits differ when they are competing only with other TP fish versus when half the fish are WR(H), given a constant density. Contrast ii tests whether there is an effect of total fish density on TP traits. Contrast iii tests whether the effect of density on TP traits depends on the identity of the competitor with whom the TP fish are competing.

Analysis C asks the same questions as analysis B, but instead focuses on WR(H) offspring and provides complementary inference.

Analysis D uses cells 3, 5, 7, and 8. For this analysis, we focus only on data from the WR fish when in competition with TP fish. Contrast i tests whether WR(H) and WR(L) differ in performance in the presence of TP fish. Contrast ii tests the effect of increased density on WR fish performance. Contrast iii tests whether the density effect differs between WR(H) and WR(L).

We did not compare the competitive ability of TP offspring when pitted against WR(L) and WR(H) offspring. This is because TP fish differed from WR(L) fish in two ways, by population of origin and by the density of the maternal environment. Thus, population of origin and maternal environment would be confounded in this comparison.

A drawback of this analysis design is the number of statistical tests on the same dependent variable; however, this approach has some virtues in that light; within any single analysis (A, B, C, or D), each contrast is independent using Type III SS in the two-way ANOVA.

All statistical analyses (described above) were carried out in SAS ® software, V 9.2, SAS Institute Inc. (Cary, NC) (ANOVA). All dependent variables were log transformed to satisfy assumptions of ANOVA.

We checked for differences in survival among treatments using logistic regression (Proc GENMOD (SAS), binomial distribution, logit link, and scaled deviance options). We found no significant differences in survival among treatments (*χ*^2^ = 10.11, *P* = 0.18) so we did not consider this further. The data on age and size at maturity presented below are only from females because very few males matured in any treatment.

## Results

### Initial offspring size

**Figure 2:**
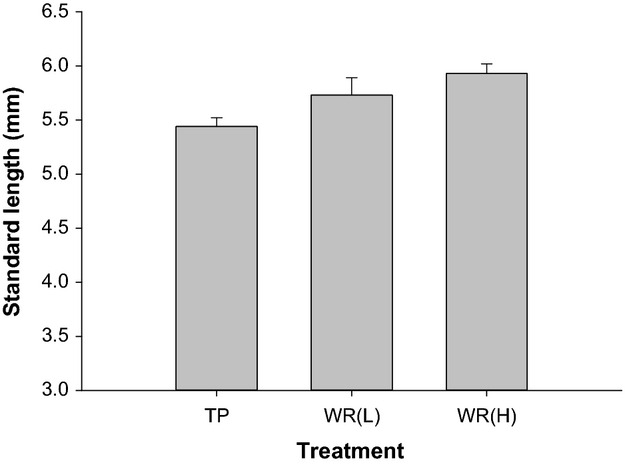
Initial standard lengths of juveniles measured prior to assignment to a particular treatment. TP = Trout Pond offspring, WR(L) = offspring of Wacissa River females held at low density, WR(H) = offspring from Wacissa River females held at high density. Shown are average values ± 1 SE.

The WR(H) offspring had the highest initial body sizes. TP offspring were the smallest, whereas the WR(L) offspring size was intermediate between the two (Fig 2). These differences in offspring size were significant, (F_2,55_ = 7.72, *P* < 0.01), with WR(H) significantly larger than TP, and WR(L) statistically indistinguishable from the others.

### Analysis A: the effect of density and population origin in single stock treatments

Quadrupling the density of offspring significantly reduced offspring growth rate by about 50% (F_1,16_ = 7.41, *P* = 0.009, Fig S2) and this effect was similar for both populations (interaction effects: F_1,16_ = 0.01, *P* = 0.93). There was not a significant interaction between population of origin and density (F_1,16_ = 0.01, *P* = 0.93).

The age at maturity for females did not appear to differ between densities (F_1,16_ = 0.33, *P* = 0.57, Fig S3), with population of origin (F_1,16_ = 0.54, *P* = 0.47), or their interaction (F_1,16_ = 0.30, *P* = 0.58). Ages at maturity were well within those estimated from otolith samples from our field sites and independent laboratory studies (unpubl. data).

Increased density had no significant effect on the size at maturity of females (F_1,16_ = 0.84, *P* = 0.36, Fig S4). Across densities, fish from W(H) stock were not significantly different for this trait (F_1,16_ = 1.12, *P* = 0.30), nor was the interaction between population and density (F_1,16_ = 0.15, *P* = 0.70).

### Analysis B: the effect of density and the identity of the competitor on Trout Pond offspring

The first contrast in this analysis was designed to test whether the identity of the competitor had an influence on TP traits. As expected, increased density significantly reduced growth rate (F_1,18_ = 13.68, *P* = 0.0007, Fig S5). We did not detect an effect of the identity of the competitor on growth rate (TP vs. WR(H) fish, F_1,18_ = 0.24, *P* = 0.63) nor did we find that the effect of density depended on the competitor type (TP vs. WR(H)), F_1,18_ = 0.66, *P* = 0.42).

**Figure 3:**
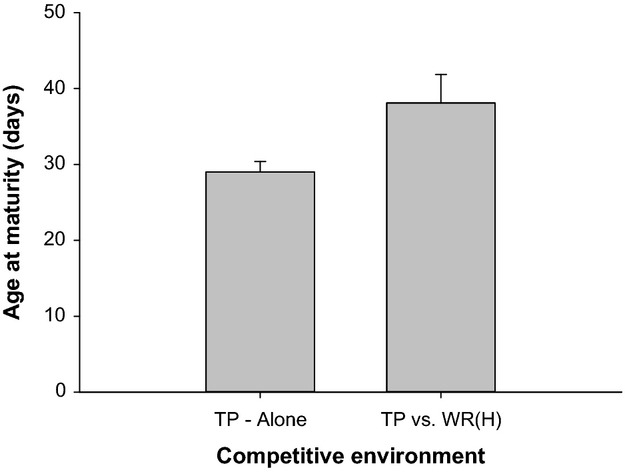
Trout Pond (TP) females delayed maturity when they were in competition with relatively large Wacissa River (WR(H)) offspring compared to when they were raised with only other TP offspring (comparisons are averaged across densities). Shown are average values + 1 SE.

Quadrupling the density did not affect the age at maturity of TP fish (F_1,16_ = 0.45, *P* = 0.51). However, compared with the values in pure TP treatments, the presence of W(H) fish caused the TP fish to delay maturity by about 24–33% (F_1,16_ = 6.10, *P* = 0.02, Fig. 3). This suggests a competitive asymmetry between TP and WR(H) fish, with the WR(H) fish being the stronger competitors. There was no evidence for an interaction between density and whether TP fish were competing with members of their own or the WR(H) population.

For size at maturity, none of the contrasts were significant (pure vs. mixed stocks: F_1,16_ = 0.03, *P* = 0.86; density: F_1,16_ = 0.27, *P* = 0.60; interaction between density and identity of the competitor: F_1,16_ = 0.00, *P* = 0.90).

### Analysis C: the effect of density and the identity of the competitor on Wacissa River offspring

This analysis was designed to test whether the identity of the competitor had an influence on any of the traits of WR(H) fish. As in all previous analyses, increased density substantially reduced WR(H) offspring growth rate (F_1,19_ = 7.25, *P* = 0.01, Table 1, Fig S6). The identity of the competitor had no significant influence on WR(H) growth rate (F_1,19_ = 0.05, *P* = 0.81), and there was no evidence for a density × competitor (WR(H) vs. TP fish) interaction (F_1,19_ = 0.01, *P* = 0.90).

**Table 1:** Means and standard errors of traits of Wacissa River (H) offspring (from Wacissa River females held at high maternal density) and Wacissa River (L) offspring when in competition with Trout Pond offspring (means for treatment groups compared in Analysis D)

	Treatment			
	
Trait	WR(H) versus TP Low Density	WR(H) versus TP High Density	WR(L) versus TP Low Density	WR(L) versus TP High Density
Growth Rate (mm/day)	0.10 (0.01)	0.06 (0.01)	0.11 (0.01)	0.07 (0.01)
Age at Maturity (days)	35 (4)	31 (1)	39 (5)	34 (4)
Size at Maturity (mm)	10.3 (0.8)	8.8 (0.5)	9.6 (0.5)	*9.9 (0.7)

* The average value shown here contains data from one replicate that may be an outlier. The average size at maturity for this treatment group without this replicate is 9.2 mm.

TP = Offspring of Trout Pond Females held at high maternal density. Data shown are average values (+ 1 SE)

**Figure 4:**
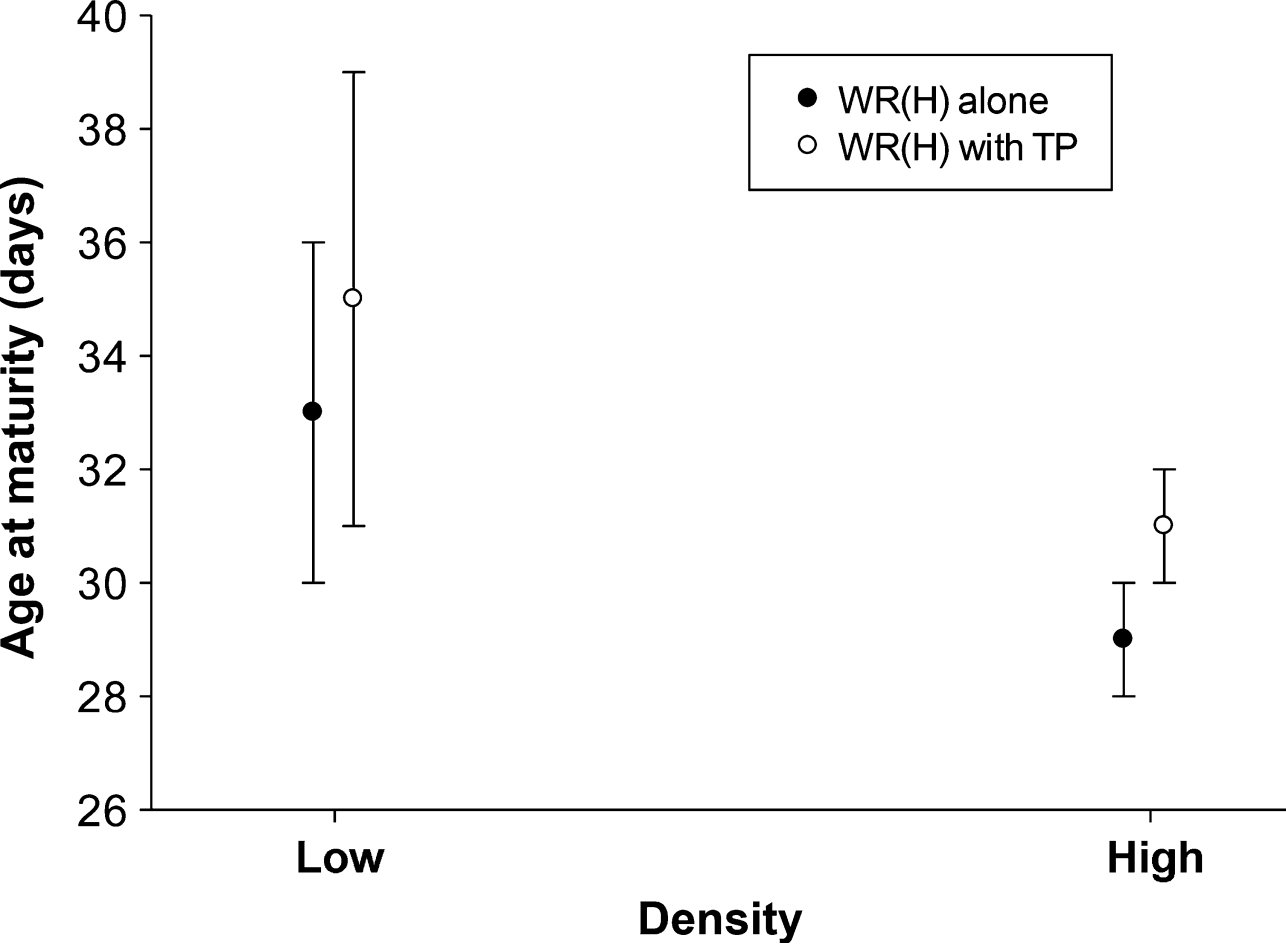
Mean age at maturity of WR(H) offspring (± 1 SE) in single stock versus mixed population treatments (means for treatment groups compared in Analysis C). TP = Offspring of Trout Pond Females held at high maternal density, WR(H) = Offspring of Wacissa River females held at high maternal density.

The patterns in age at maturity of WR(H) fish when alone or with TP fish were slightly different than those seen in TP fish. First, increased density actually decreased age at maturity as opposed to the lack of an effect of density seen in the TP fish (Fig. 4). Second, they matured only slightly (and nonsignificantly) later in the presence of TP fish, compared with the pronounced delay in TP fish in the presence of WR(H) fish (compare values in Table 1, Figs 3 and 4). None of the contrasts was statistically significant (pure vs. mixed stocks: F_1,17_ = 0.10, *P* = 0.76; density: F_1,17_ = 1.48, *P* = 0.23; interaction between density and identity of the competitor: F_1,17_ = 0.0, *P* = 0.98). These results are consistent with WR(H) fish being stronger competitors than TP fish.

The patterns in size at maturity for WR(H) fish reflected those seen in the TP fish, in which none of the effects were statistically significant (effect of competitor: F_1,17_ = 0.01, *P* = 0.91, density: F_1,17_ = 3.13, *P* = 0.08, interaction: F_1,17_ = 0.28, *P* = 0.60, Table 1, Fig S7).

### Analysis D: the effect of relative size of Wacissa River fish on competitive ability

Analysis D focused on traits of Wacissa River offspring reared from the two maternal environments (WR[H] and WR[L]) and placed in competition with Trout Pond fish. This analysis was designed to reveal how different initial sizes and prenatal experiences of Wacissa River offspring influence growth, age and size at maturity in competition against a common “tester” stock (TP) at low and high density.

The average values for all traits are shown in Table 1. In the first contrast, we found no significant differences in growth rate between the relatively larger WR(H) offspring and the smaller WR(L) offspring (F_1,19_ = 1.48, *P* = 0.23). In the second contrast, as in our earlier analyses, we did find a significant influence of density on the growth rate of offspring (F_1,19_ = 11.90, *P* = 0.002). The final contrast that asked if the effect of density differed on WR(H) versus WR(L) fish was not significant (F_1,19_ = 0.01, *P* = 0.93).

For age at maturity, none of the contrasts was significant (WR[H] vs. WR[L]: F_1,17_ = 1.03, *P* = 0.32 density: F_1,17_ = 1.34, *P* = 0.26; interaction between density and offspring size/maternal environment: F_1,17_ = 0.0, *P* = 0.98).

For the size at maturity, as was the case for age at maturity, none of the contrasts was significant (WR[H] vs. WR[L]: F_1,17_ = 0.17, *P* = 0.68; density: F_1,17_ = 1.16, *P* = 0.29; interaction: F_1,17_ = 1.79, *P* = 0.19).

## Discussion

There were two main outcomes of this study. First, all of the offspring grew significantly more slowly in the higher density regardless of their identity and the identity of the competitor. Second, fish that were significantly larger at birth and were derived from a population that experiences extremely high densities (WR[H] fish) were better competitors, in the sense that, when paired with fish from the low-density population (TP), they caused a delay in the onset of sexual maturity in TP females compared with the onset in a homogeneous cohort of TP fish.

### Density effects in single stock treatments

Quadrupling density decreased juvenile growth rates by about 50%, regardless of the population. The lack of a difference between populations in response to density is striking because fish in Trout Pond experience densities that are consistently two or more orders of magnitude lower than those at the Wacissa River (Leips and Travis 1999); this is a chronic difference that might lead one to expect some density-dependent adaptation. Per capita resource availability in the natural populations remains unknown, and so it is possible that the resources available per individual are similar in the two populations; however, this seems unlikely given what we know about them (unpubl. data from otoliths, etc.). An additional caveat is that some aspect of the laboratory treatment (e.g., the use of flake food) may have masked population-specific responses of growth rate to density. Future work on this system could test these possibilities by using more natural food types (e.g., zooplankton) across a wider range of per capita resource levels.

There were no obvious effects of density on the size at sexual maturity for females. There were also no significant effects of density on the age at maturity for females, although there was evidence of a population-specific response to density. WR fish appeared to mature earlier at higher density while maturation rates of TP fish appeared unaffected by increased density in single stock treatments. We discuss these potential population-specific responses to density in more detail below. The lack of an effect of density on the age at maturity is surprising given its effect on early growth rates and the normally depressant effects of density on development rates of ectothermic vertebrates (Wilbur 1980). However, because we only measured age at maturity once a week, if the treatments produced differences of less than 1 week, we would not have detected them.

### Equivalence of competitors? Single stock versus mixed stock comparisons

With respect to initial growth rates, our results indicate that fish from both populations are equivalent competitors. In contrast, we found evidence of competitive asymmetry effects on the age at maturity, between fish from TP and WR. The observed patterns in age at maturity suggest that when placed in mixed stock treatments, fish from the two populations have different norms of reaction to density. If anything, WR(H) offspring appear to mature slightly (although not significantly) earlier when exposed to increased densities in every case, regardless of the identity of the competitor. In contrast, as density increased, offspring from TP delayed maturity when mixed with WR(H), but matured at the same age when in competition with members of their own population; we also note that there was no effect of density on growth rate or size at maturity of TP fish when compared across these treatments. So if growth rates were comparable but age at maturity differed, there should have been an effect on the size at maturity. This means that either the effects of WR fish on the age at maturity of TP offspring are overestimated by the sampling method we used, or that growth rates of developing females in the different treatments diverged after growth rate estimates were made (after the 2-week period). Another possibility is that small differences in growth rate may produce large differences in the age at maturity. There is no way to distinguish between these hypotheses with the current data, but these possibilities would be useful to explore in future experiments.

While competition may explain the density-dependent depressant effects of WR fish on the age at maturity of TP fish, the WR fish accelerated the age at maturity with increasing density, regardless of the identity of the competitor. This suggests that density-dependent competition is not the sole factor influencing this trait in WR fish. One possible explanation is that density-dependent social effects influenced the age at maturity in WR females. Social effects on age at maturity are commonly reported in fish (Borowsky 1978; Bushman and Burns 1994; Danylchuk and Tonn 2001; Aday et al. 2003; Walling et al. 2007) and are attributed to a variety of potential mechanisms including the existence of dominance hierarchies, aggressive interactions, and chemical signaling (Aday et al. 2003). As WR fish typically experience much higher densities than TP fish, it is possible that these populations have evolved different sensitivities to social interactions that influence density-dependent maturation rates.

### Fitness consequences of differences in offspring size

Our results indicate that under competitive conditions, for *H. formosa*, it is always better to be relatively bigger than your competitor. The larger offspring from the Wacissa River were never worse competitors than TP offspring and, under some conditions, were clearly superior (TP delayed maturity when WR[H] were present, compared to TP alone and, conversely, WR[H] were unaffected by presence of TP). As delayed maturity increases time to first reproduction, all else being equal, delaying the age at sexual maturity should reduce fitness. Along these same lines, we note that the relatively larger WR(H) offspring matured earlier than the smaller WR(L) offspring when they were mixed with TP (Table 1). Although not significant, the trend reinforces the hypothesis that WR(H) offspring are better competitors than the smaller WR(L) offspring, and that larger offspring size may allow earlier maturation than smaller offspring size. In this light, we propose that the increased size of offspring produced by females experiencing high density (Leips et al. 2009, this study) represents an adaptive, plastic response that increases offspring fitness in high density, competitive environments. Whether the fitness advantage of being relatively larger results from competitive superiority and/or some other mechanism such as increased aggressiveness toward relatively smaller individuals remains an open question.

Our results raise an important question that remains the subject of ongoing debate: if increased size of offspring at birth is adaptive, for whom is it so (Wolf and Wade 2001; Marshall and Uller 2007; Uller and Pen 2011)? More precisely, is this effect an adaptation of mothers or offspring? Maternal adaptation is the more familiar hypothesis (Marshall and Uller 2007). This argument has two parts. First, in the context of this study, crowding has selected for females to allocate more resources to offspring because crowding signals resource shortage and a shortened lifespan of adults. Life history theory predicts that an increase in the mortality rates of older age-classes should select for an increase in investment in reproduction at ages before the increased mortality (Charlesworth 1994). The same logic applied to plasticity in female investment argues that when environmental cues indicate that the risk of mortality for an individual is about to increase, she should increase her investment in reproduction. Because *H. formosa* are matrotrophs and cannot quickly change their fecundity in response to resource level availability, we might expect the change in investment be reflected in offspring sizes rather than number (Pollux and Reznick 2011). Second, females may use social cues as an indicator of future high levels of crowding and resource shortages, allowing them to compensate for deteriorating conditions. Isolated *H. formosa* females receiving reduced food levels have been reported to produce smaller offspring (Reznick et al. 1996; but see Travis et al. 1987). This could be maladaptive in situations where there is selection for a large body size. Therefore, crowding may offer a cue about resource shortages that acts in advance of the actual effect of that shortage. This could allow females to adjust their investment almost immediately so that they can keep their offspring at a relatively constant size despite falling per capita food levels. This would be an example of a “counter gradient” phenomenon (Levins 1969; Conover and Schultz 1995); in nature, increased crowding would be associated with lower per capita food and the response we have documented in females may act to keep offspring size near the optimal level.

Increased size at birth could also/instead be an adaptation of offspring. Being larger can not only benefit offspring fitness, especially in competitive conditions, if larger size enhances resource acquisition during the juvenile stage this could also enhance the reproductive fitness of adults (Lindstrom 1999; Auer 2010). We know that offspring can influence maternal investment (Schrader and Travis 2009), and so we can posit that offspring can coerce greater maternal investment when mothers experience higher densities. This hypothesis requires 1) that there be a physiological cue associated with higher density, 2) that the cue passes through the placenta in the female ovary to the embryo, and 3) that the embryo responds appropriately. With respect to 1), a common response to crowding in other vertebrates is increased secretion of cortisol (Rotllant and Tort 1997; Ramsay et al. 2009). It remains to be demonstrated whether female *H. formosa* respond similarly and whether embryos can seize upon this cue and increase production of compounds like *IGF-2,* which is associated with increased resource transfer from mother to embryo, to coerce greater investment from their mothers. If embryos do not respond to elevated cortisol, females might direct resources away from reproduction, a commonly observed trend in egg laying fish (McCormick 2006; Mileva et al. 2011). There is evidence that even exposure of unfertilized eggs to elevated but physiologically relevant levels of cortisol can influence behavioral and morphological traits of juvenile fish (Sloman 2010) so our hypothesis is plausible.

Our results suggest that genetically based differences in offspring size between populations are maintained in part by the selective advantage of being relatively large in a competitive environment (e.g., the high-density population of the Wacissa River). This interpretation is consistent with studies across a broad range of taxa that have found that larger propagule sizes are selectively favored in competitive environments (Hutchings 1991; Guisande 1993; Sinervo et al. 2000; Marshall et al. 2006; Allen et al. 2008). We recognize that additional ecological factors like predation may contribute to the divergence in offspring size between these populations. Indeed, in a 10 year study of life history variation in about a dozen populations of *H. formosa* (including the TP and WR populations), Schrader and Travis (2012) found that increased density and decreased predation risk were each independently associated with larger offspring sizes. This result indicates that while density is not the only likely agent of selection on offspring size, density is acting independently of the action of predation as an agent of selection.

A number of mechanisms can contribute to the competitive advantage of larger size; larger individuals may be more capable of sustained foraging activity (Parkos and Wahl 2010), they may be more effective at rapidly harvesting scarce algal resources, be able to eat a greater diversity of food types, or be more likely to prevail in competitive interactions for resources (e.g., because of higher burst speeds (Segers and Taborsky 2011)). Size can be an important factor determining the outcome of aggressive encounters (e.g., Farr 1989). Whether large size is genetically correlated with another trait such as evolved differences in aggressive behavior (e.g., Herczeg and Valimaki 2011) or egg composition (e.g., Ruuskanen et al. 2011) that improves competitive ability is not known.

While being large may also be advantageous in noncompetitive environments, because of the tradeoff between offspring size and offspring number, the relative advantage of having large offspring in noncompetitive environments may not offset the resultant reduction in parental fitness due to the decrease in offspring number. This may well be the case for the TP population. Information on the size-specific mortality rates of juveniles in natural populations that differ in density could help to test this idea. Additional information on the relationship between offspring size and fitness across a range of densities (cf. Winn and Miller 1995) could also contribute to our understanding of the adaptive significance of patterns of population density and offspring size in natural populations.

## Appendix

**Table A1:** The experimental matrix of treatment combinations. Numbers in each cell refer to designations for contrasts between different treatments. Densities: 1/2*N* = 4 individuals, *N* = 8 individuals and 4*N* = 32 individuals. T = Trout Pond offspring, W = Wacissa River offspring (H = offspring of females maintained at high density, L = offspring from females maintained at low density)

	Density of Trout Pond Offspring				
	
Density of Wacissa River Offspring	0	½ *N*	*N*	2*N*	4*N*
0	…	…	1	…	2
½*N* (H)	…	3	…	…	…
*N* (H)	4	…	…	…	…
2*N* (H)	…	…	…	5	…
4*N* (H)	6	…	…	…	…
½*N* (L)	…	7	…	…	…
2*N* (L)	…	…	…	8	…

## Supporting Information

**Figure S1.** A photo of one cattle tank with the mesh bags suspended in the tank. Each bag contained one replicate of each of the 8 treatments.

**Figure S2.** Mean growth rates of offspring (± 1 SE) from single stock treatments (means for treatment groups compared in Analysis A). TP = Offspring of Trout Pond Females held at high maternal density, WR(H) = Offspring of Wacissa River females held at high maternal density.

**Figure S3.** Mean age at maturity of offspring (± 1 SE) from single stock treatments (means for treatment groups compared in Analysis A). TP = Offspring of Trout Pond Females held at high maternal density, WR(H) = Offspring of Wacissa River females held at high maternal density.

**Figure S4.** Mean size at maturity of offspring (± 1 SE) from single stock treatments (means for treatment groups compared in Analysis A). TP = Offspring of Trout Pond Females held at high maternal density, WR(H) = Offspring of Wacissa River females held at high maternal density.

**Figure S5.** Mean growth rates of TP offspring (± 1 SE) single stock versus mixed stock treatments (means for treatment groups compared in Analysis B). TP = Offspring of Trout Pond Females held at high maternal density, WR(H) = Offspring of Wacissa River females held at high maternal density.

**Figure S6.** Mean growth rates of WR(H) offspring (± 1 SE) single versus mixed stock treatments (means for treatment groups compared in Analysis C). TP = Offspring of Trout Pond Females held at high maternal density, WR(H) = Offspring of Wacissa River females held at high maternal density.

**Figure S7.** Mean size at maturity of WR(H) offspring (± 1 SE) single versus mixed stock treatments (means for treatment groups compared in Analysis C). TP = Offspring of Trout Pond Females held at high maternal density, WR(H) = Offspring of Wacissa River females held at high maternal density.

**Table S1.** The top four rows reexpress the information in **Table A1.** The top three rows indicate the identity and density of fish in that treatment level (Population MD = population and maternal density) and a fourth row that indicates the cell number used in Table A1 for that combination of TP (H), WR(H), or WR (L) fish. The remainder of the table is a set of contrasts among treatment levels that describe four two-way analyses of variance with different combinations of levels used in each analysis. The four analyses are designated by the Roman letters A–D. Within any single “analysis” (A, B, C, or D), there are three contrasts designated i, ii, and iii. The first two contrasts, i and ii, test for main effects and the third contrast, iii, tests for the interaction between two main effects. Analyses and hypotheses tested contrasts within each analysis are described in the text.

## References

[b1] Aday DD, Wahl DH, Philip DP (2003). A mechanism for social inhibition of sexual maturation in bluegill. J. Fish Biol.

[b2] Allen RM, Buckley YM, Marshall DJ (2008). Offspring size plasticity in response to intraspecific competition: an adaptive maternal effect across life-history stages. Am. Nat.

[b3] Amat JA, Fraga RM, Arroyo GM (2001). Intraclutch egg-size variation and offspring survival in the Kentish Plover *Charadrius alexandrinus*. Ibis.

[b4] Auer SK (2010). Phenotypic plasticity in adult life-history strategies compensates for a poor start in life in Trinidadian guppies (*Poecilia reticulata*. Am. Nat.

[b5] Baer CE (1998). Species-wide population structure in a southeastern US freshwater fish, *Heterandria formosa*: gene flow and biogeography. Evolution.

[b6] Bagenal TB (1969). Relationship between egg size and fry survival in brown trout *Salmo trutta*. J. Fish Biol.

[b7] Bashey F (2006). Cross-generational environmental effects and the evolution of offspring size in the Trinidadian guppy *Poecilia reticulata*. Evolution.

[b8] Bashey F (2008). Competition as a selective mechanism for larger offspring size in guppies. Oikos.

[b9] Berven KA, Chadra BG (1988). The relationship among egg size, density and food level on larval development in the wood frog (*Rana sylvatica*. Oecologia.

[b10] Borowsky RL (1978). Social inhibition of maturation in natural populations of *Xiphophorus variatus* (Pisces: Poecliidae). Science.

[b11] Brockelman WY (1975). Competition, the fitness of offspring, and optimal clutch size. Am. Nat.

[b12] Burns CW (1995). Effects of crowding and different food levels on growth and reproductive investment of *Daphnia*. Oecologia.

[b13] Bushman PJ, Burns JR (1994). Social control of male sexual maturation in the swordtail characin, *Corynopoma riisei*. J. Fish Biol.

[b14] Charlesworth B (1994). Evolution in Age-Structured Populations.

[b15] Collin R, Salazar MZ (2010). Temperature-mediated plasticity and genetic differentiation in egg size and hatching size among populations of Crepidula (Gastropoda: Calyptraeidae). Biol. J. Linn. Soc.

[b16] Conover DO, Schultz ET (1995). Phenotypic similarity and the evolutionary significance of countergradient variation. Trends Ecol. Evol.

[b17] Constanz GD, Meffe GK, Snelson FF (1989). Reproductive biology of poeciliid fishes. Ecology and Evolution of Livebearing Fishes.

[b18] Danylchuk AJ, Tonn WM (2001). Effects of social structure on reproductive activity in male fathead minnows (*Pimephales promelas*. Behav. Ecol.

[b19] Dunson WA, Travis J (1991). The role of abiotic factors in community organization. Am. Nat.

[b20] Einum S, Fleming IA (1999). Maternal effects of egg size in brown trout (*Salmo trutta*): norms of reaction to environmental quality. Proc. Roy. Soc. Lon. Ser. B Biol. Sci.

[b21] Farr JA, Meffe GK, Snelson FF (1989). Sexual selection and secondary sexual differentiation in Poeciliids: determinants of male mating success and the evolution of female choice. Ecology and Evolution of Livebearing Fishes (Poeciliidae).

[b22] Fox CW, Czesak ME (2000). Evolutionary ecology of progeny size in arthropods. Annu. Rev. Entomol.

[b23] Fox CW, Thakar MS, Mousseau TA (1997). Egg size plasticity in a seed beetle: an adaptive maternal effect. Am. Nat.

[b24] Fraser EA, Renton RM (1940). Observation on the breeding and development of the viviparous fish, *Heterandria formosa*. Q. J. Microsc. Sci.

[b25] Gliwicz ZM, Guisande C (1992). Family planning in *Daphnia*: resistance to starvation in offspring born to mothers grown at different food levels. Oecologia.

[b26] Guisande C (1993). Reproductive strategy as population-density varies in *Daphnia magna* (Cladocera). Freshw. Biol.

[b27] Harper JL (1977). Population Biology of Plants.

[b28] Herczeg G, Valimaki KK (2011). Intraspecific variation in behaviour: effects of evolutionary history, ontogenetic experience and sex. J. Evol. Biol.

[b29] Hutchings JA (1991). Fitness consequences of variation in egg size and food abundance in brook trout *Salvelinus fontinalis*. Evolution.

[b30] Jacobs MW, Sherrard KM (2010). Bigger is not always better: offspring size does not predict growth or survival for seven ascidian species. Ecology.

[b31] Janzen FJ, Warner DA (2009). Parent-offspring conflict and selection on egg size in turtles. J. Evol. Biol.

[b32] Kaplan RH (1992). Greater maternal investment can decrease offspring survival in the frog *Bombina orientalis*. Ecology.

[b33] Kawecki TJ (1995). Adaptive plasticity of egg size in response to competition in the cowpea weevil, *Callosobruchus maculatus* (Coleoptera:Bruchidae). Oecologia.

[b34] Kindsvater HK, Alonzo SH, Mangel M, Bonsall MB (2010). Effects of age- and state-dependent allocation on offspring size and number. Evol. Ecol. Res.

[b35] Leips J, Travis J (1999). The comparative expression of life-history traits and its relationship to the numerical dynamics of four populations of the least killifish. J. Anim. Ecol.

[b36] Leips JJ, Travis J, Rodd FH (2000). Genetic influences on experimental population dynamics of the least killifish. Ecol. Monogr.

[b37] Leips J, Baril CT, Rodd FH, Reznick DN, Bashey F, Visser GJ (2001). The suitability of calcein to mark poeciliid fish and a new method of detection. Trans. Am. Fish. Soc.

[b38] Leips J, Richardson JML, Rodd FH, Travis J (2009). Adaptive maternal adjustments of offspring size in response to conspecific density in two populations of the least killifish, *Heterandria formosa*. Evolution.

[b39] Levins R (1969). Thermal acclimation and heat resistance in *Drosophila* species. Am. Nat.

[b40] Lindstrom J (1999). Early development and fitness in birds and mammals. Trends Ecol. Evol.

[b41] Lloyd DG (1987). Selection of offspring size at independence and other size- versus-number strategies. Am. Nat.

[b42] Marshall DJ, Keough MJ (2007). The evolutionary ecology of offspring size in marine invertebrates. Adv. Mar. Biol.

[b43] Marshall DJ, Keough MJ (2009). Does interspecific competition affect offspring provisioning?. Ecology.

[b44] Marshall DJ, Uller T (2007). When is a maternal effect adaptive?. Oikos.

[b45] Marshall DJ, Cook CN, Emlet RB (2006). Offspring size effects mediate competitive interactions in a colonial marine invertebrate. Ecology.

[b46] McCormick MI (2006). Mothers matter: crowding leads to stressed mothers and smaller offspring in marine fish. Ecology.

[b47] McGinley MA, Temme DH, Geber MA (1987). Parental investment in offspring in variable environments: theoretical and empirical considerations. Am. Nat.

[b48] Mileva VR, Gilmour KM, Balshine S (2011). Effects of maternal stress on egg characteristics in a cooperatively breeding fish. Comp. Biochem. Physiol. A Mol. Integr. Physiol.

[b49] Parker GA, Begon M (1986). Optimal egg size and clutch size: effects of environment and maternal phenotype. Am. Nat.

[b50] Parkos JJ, Wahl DH (2010). Influence of body size and prey type on the willingness of age-0 fish to forage under predation risk. Trans. Am. Fish. Soc.

[b51] Perrin N (1989). Population density and offspring size in the cladoceran *Simocephalus vetulus*. Funct. Ecol.

[b52] Pollux BJA, Reznick DN (2011). Matrotrophy limits a female's ability to adaptively adjust offspring size and fecundity in fluctuating environments. Funct. Ecol.

[b53] Ramsay JM, Feist GW, Varga ZM, Westerfield M, Kent ML, Schreck CB (2009). Whole-body cortisol response of zebrafish to acute net handling stress. Aquaculture.

[b54] Reed WL, Clark ME, Vleck CM (2009). Maternal effects increase within- family variation in offspring survival. Am. Nat.

[b55] Reznick DN (1982). The impact of predation on life-history evolution in Trinidadian guppies - genetic-basis of observed life-history patterns. Evolution.

[b56] Reznick D, Endler JA (1982). The impact of predation on life history evolution in Trinidadian guppies (*Poecilia reticulata*. Evolution.

[b57] Reznick D, Yang AP (1993). The influence of fluctuating resources on life history: patterns of allocation and plasticity in female guppies. Ecology.

[b58] Reznick DA, Bryga H, Endler JA (1990). Experimentally induced life- history evolution in a natural population. Nature.

[b59] Reznick D, Callahan H, Llauredo R (1996). Maternal effects on offspring quality in poeciliid fishes. Am. Zool.

[b60] Reznick D, Butler IV MJ, Rodd H (2001). Life-history evolution in guppies VII. The comparative ecology of high- and low-predation environments. Am. Nat.

[b61] Richardson JML, Gunzburger MS, Travis J (2006). Variation in predation pressure as a mechanism underlying differences in numerical abundance between populations of the poeciliid fish *Heterandria formosa*. Oecologia.

[b62] Riesch R, Tobler M, Plath M, Schlupp I (2009). Offspring number in a livebearing fish (*Poecilia mexicana*, Poeciliidae): reduced fecundity and reduced plasticity in a population of cave mollies. Environ. Biol. Fishes.

[b63] Rius M, Turon X, Dias GM, Marshall DJ (2010). Propagule size effects across multiple life-history stages in a marine invertebrate. Funct. Ecol.

[b64] Rotllant J, Tort L (1997). Cortisol and glucose responses after acute stress by net handling in the sparid red porgy previously subjected to crowding stress. J. Fish Biol.

[b65] Ruuskanen S, Siitari H, Eeva T, Belskii E, Jarvinen A, Kerimov A (2011). Geographical variation in egg mass and egg content in a passerine bird. PLoS ONE.

[b66] Schrader M, Travis J (2005). Population differences in pre- and post- fertilization offspring provisioning in the Least Killifish, *Heterandria formosa*. Copeia.

[b67] Schrader M, Travis J (2009). Do embryos influence maternal investment? Evaluating maternal-fetal coadaptation and the potential for parent- offspring conflict in a placental fish. Evolution.

[b68] Schrader M, Travis J (2012). Assessing the roles of population density and predation risk in the evolution of offspring size in populations of a placental fish. Ecol. Evol.

[b69] Segers FH, Taborsky B (2011). Juvenile exposure to predator cues induces a larger egg size in fish. Proc. R. Soc. B.

[b70] Sibley R, Calow P (1983). An integrated approach to life-cycle evolution using selective landscapes. J. Theor. Biol.

[b71] Sibly RM, Calow P (1986). Physiological Ecology of Animals: An Evolutionary Approach.

[b72] Sinervo B, Svensson E, Comendant T (2000). Density cycles and an offspring quantity and quality game driven by natural selection. Nature.

[b73] Sloman KA (2010). Exposure of ova to cortisol pre-fertilisation affects subsequent behaviour and physiology of brown trout. Horm. Behav.

[b74] Smith CC, Fretwell SD (1974). The optimal balance between size and number of offspring. Am. Nat.

[b75] Soucy S, Travis J (2003). Multiple paternity and population genetic structure in natural populations of the poeciliid fish, *Heterandria formosa*. J. Evol. Biol.

[b76] Stanton ML (1984). Seed variation in wild radish: effect of seed size on components of seedling and adult fitness. Ecology.

[b77] Stearns S (1976). Life-history tactics: a review of the ideas. Q. Rev. Biol.

[b78] Tessier AJ, Consolati NL (1989). Variation in offspring size in Daphnia and consequences for individual fitness. Oikos.

[b79] Travis J, Farr JA, Henrich S, Cheong RT (1987). Testing theories of clutch overlap with the reproductive ecology of *Heterandria formosa*. Ecology.

[b80] Uller T, Pen I (2011). A theoretical model of the evolution of maternal effects under parent-offspring conflict. Evolution.

[b81] Walling CA, Royle NJ, Metcalfe NB, Lindstrom J (2007). Green swordtails alter their age at maturation in response to the population level of male ornamentation. Biol. Lett.

[b82] Warner SC, Dunson WA, Travis J (1991). Interaction of pH, density, and priority effects on the survivorship and growth of two species of hylid tadpoles. Oecologia.

[b83] Wilbur HM (1980). Complex life cycles. Annu. Rev. Ecol. Syst.

[b84] Winkler DW, Wallin K (1987). Offspring size and number: a life history model linking effort per offspring and total effort. Am. Nat.

[b85] Winn AA, Miller TE (1995). Effect of density on the magnitude of directional selection on seed mass and emergence time in *Plantago wrightiana* Dcne (Plantaginaceae). Oecologia.

[b86] Wolf JB, Wade MJ (2001). On the assignment of fitness to parents and offspring: whose fitness is it and when does it matter?. J. Evol. Biol.

[b87] Zar JH (2009). Biostatistical Analysis.

