# Supplementary figures and images for: The adaptive significance of population differentiation in offspring size of the least killifish, Heterandria formosa
Source: Ecol Evol. 2013 Mar 5;3(4):948–60. doi: 10.1002/ece3.509 (PMC3631406; doi:10.1002/ece3.509)

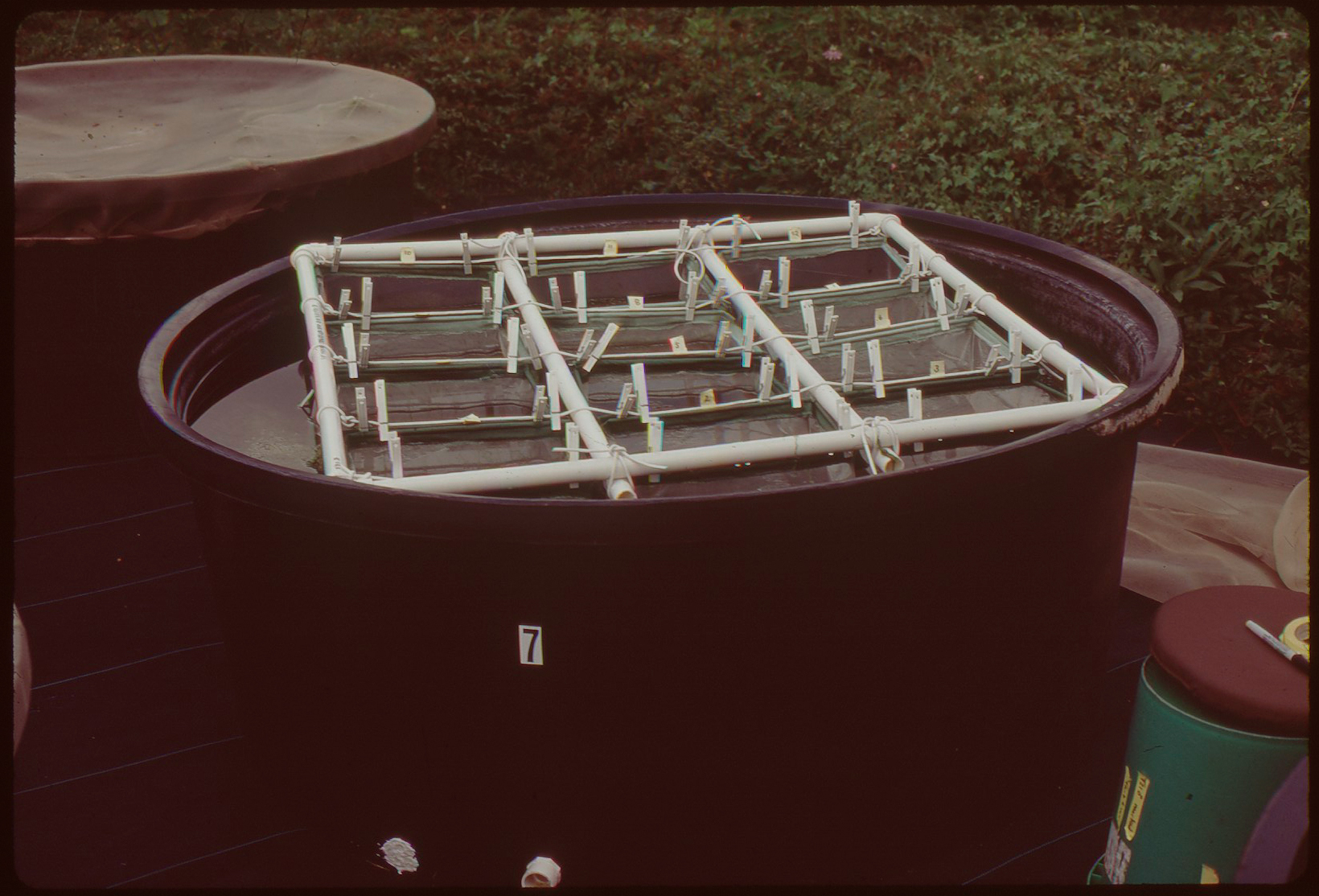

Supplement: Supplementary file 1 [file ece30003-0948-SD1.jpg]
