# Supplementary material for: The adaptive significance of population differentiation in offspring size of the least killifish, Heterandria formosa
Source: Ecol Evol. 2013 Mar 5;3(4):948–60. doi: 10.1002/ece3.509 (PMC3631406; doi:10.1002/ece3.509)

Supplemental Materials

**Figure Legends for Figures S2-S7:**

Figure S2**.** Mean growth rates of offspring (± one standard error) from single stock treatments (means for treatment groups compared in Analysis A). TP = Offspring of Trout Pond Females held at high maternal density, WR(H) = Offspring of Wacissa River females held at high maternal density.

Figure S3**.** Mean age at maturity of offspring (± one standard error) from single stock treatments (means for treatment groups compared in Analysis A). TP = Offspring of Trout Pond Females held at high maternal density, WR(H) = Offspring of Wacissa River females held at high maternal density.

Figure S4**.** Mean size at maturity of offspring (± one standard error) from single stock treatments (means for treatment groups compared in Analysis A). TP = Offspring of Trout Pond Females held at high maternal density, WR(H) = Offspring of Wacissa River females held at high maternal density.

Figure S5. Mean growth rates of TP offspring (± one standard error) single stock vs. mixed stock treatments (means for treatment groups compared in Analysis B). TP = Offspring of Trout Pond Females held at high maternal density, WR(H) = Offspring of Wacissa River females held at high maternal density.

Figure S6. Mean growth rates of WR(H) offspring (± one standard error) single stock vs. mixed stock treatments (means for treatment groups compared in Analysis C). TP = Offspring of Trout Pond Females held at high maternal density, WR(H) = Offspring of Wacissa River females held at high maternal density.

Figure S7. Mean size at maturity of WR(H) offspring (± one standard error) single stock vs. mixed stock treatments (means for treatment groups compared in Analysis C). TP = Offspring of Trout Pond Females held at high maternal density, WR(H) = Offspring of Wacissa River females held at high maternal density.

Figure S2


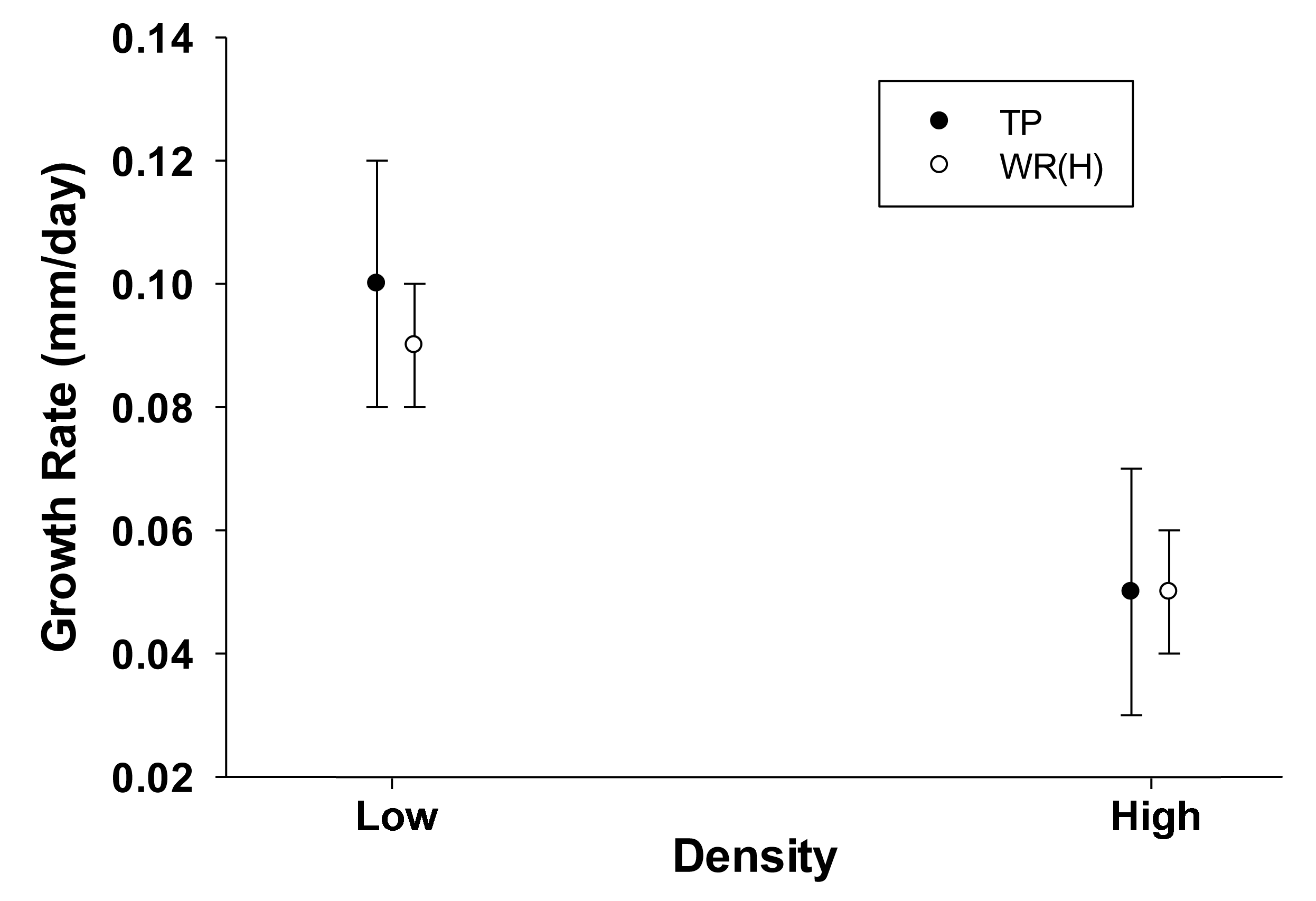


Figure S3


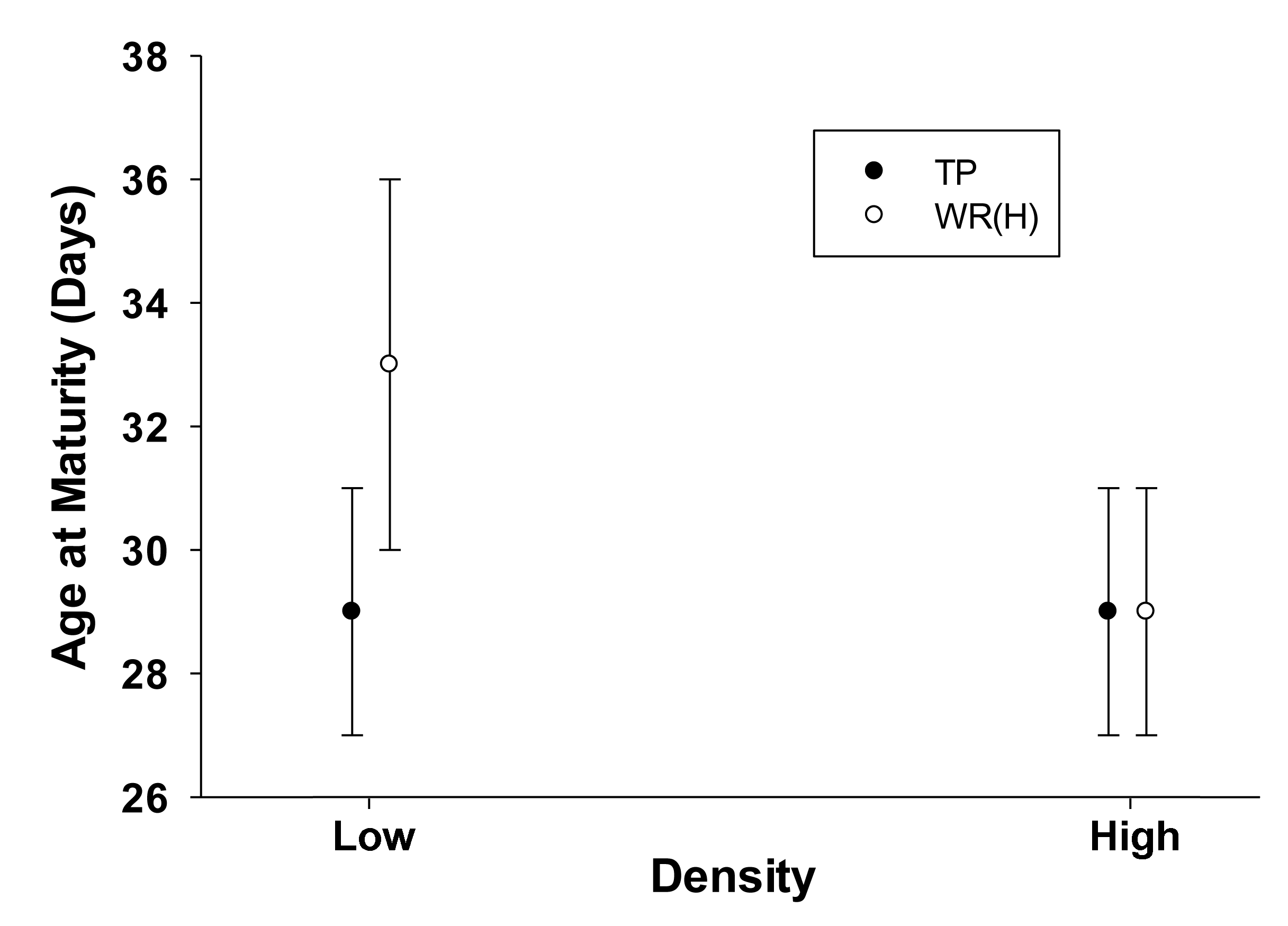


Figure S4


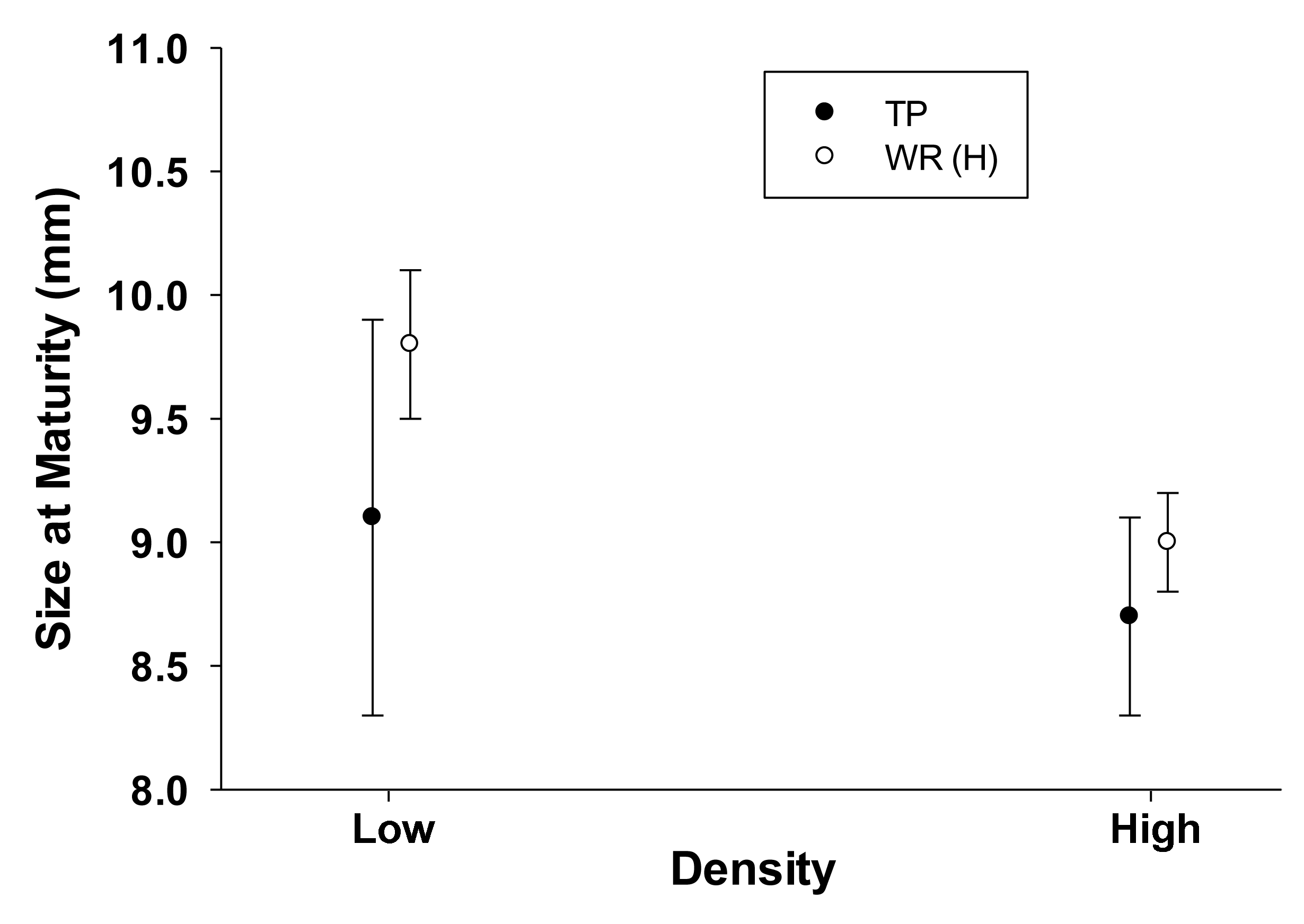


Figure S5


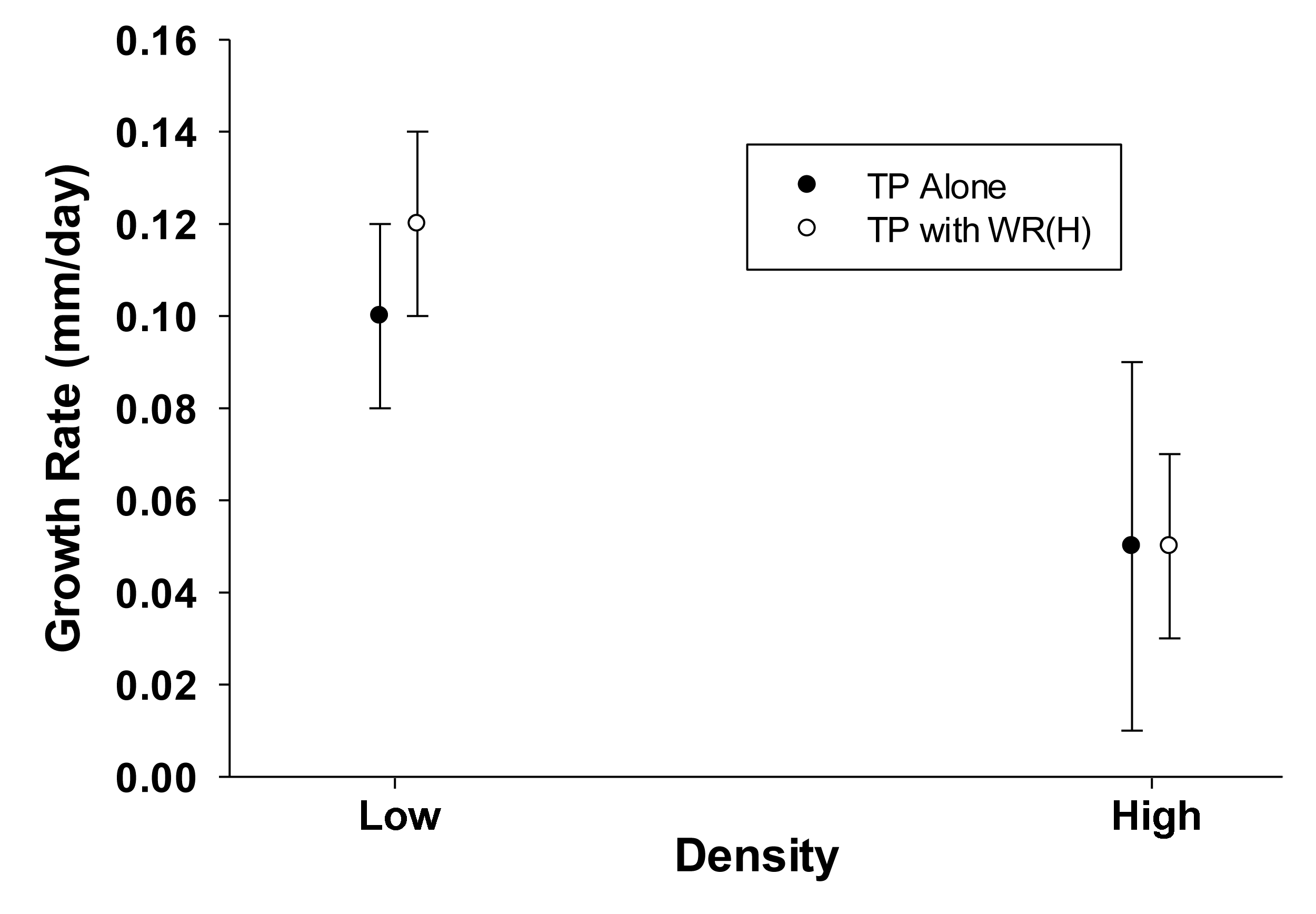


Figure S6


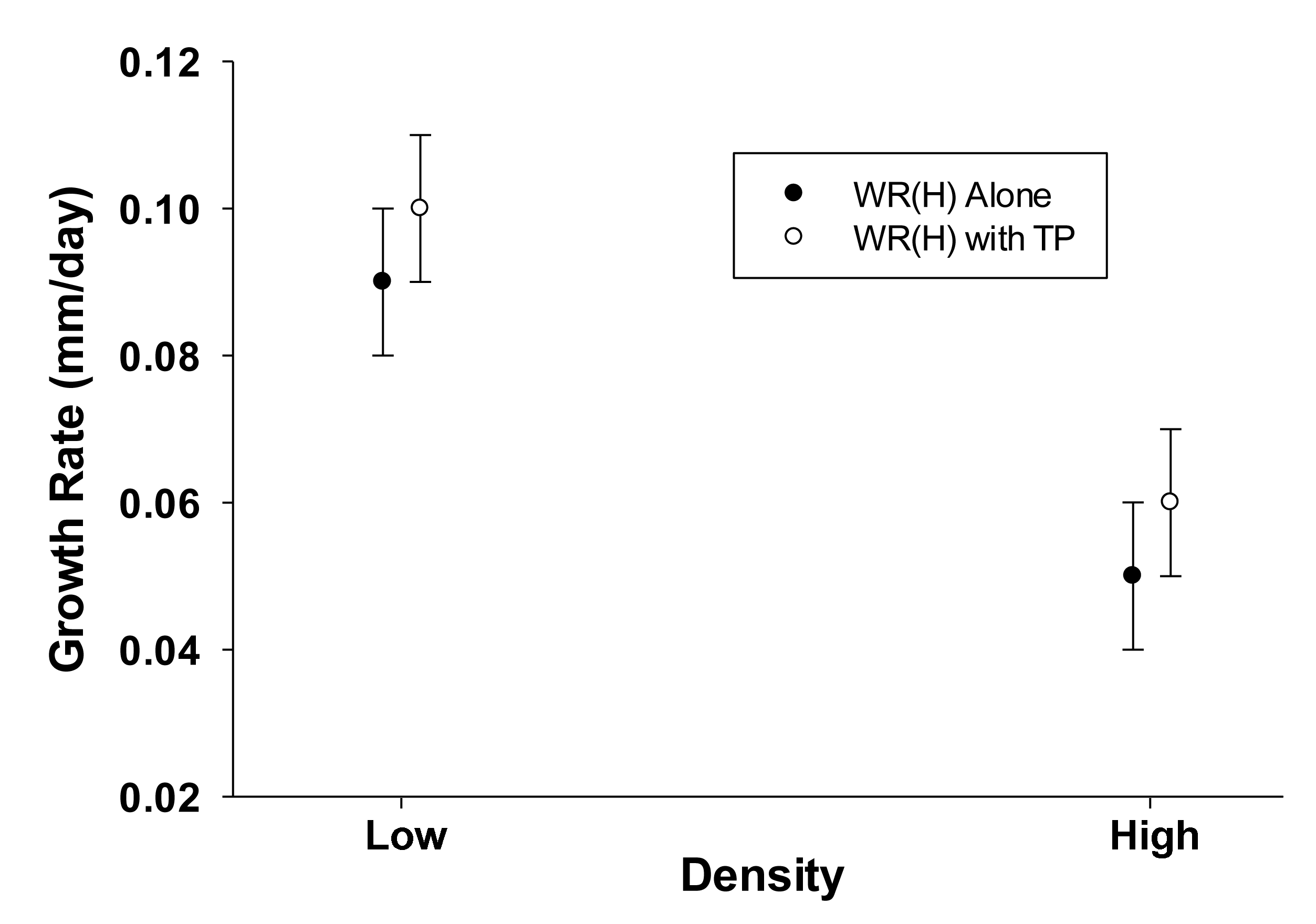


Figure S7


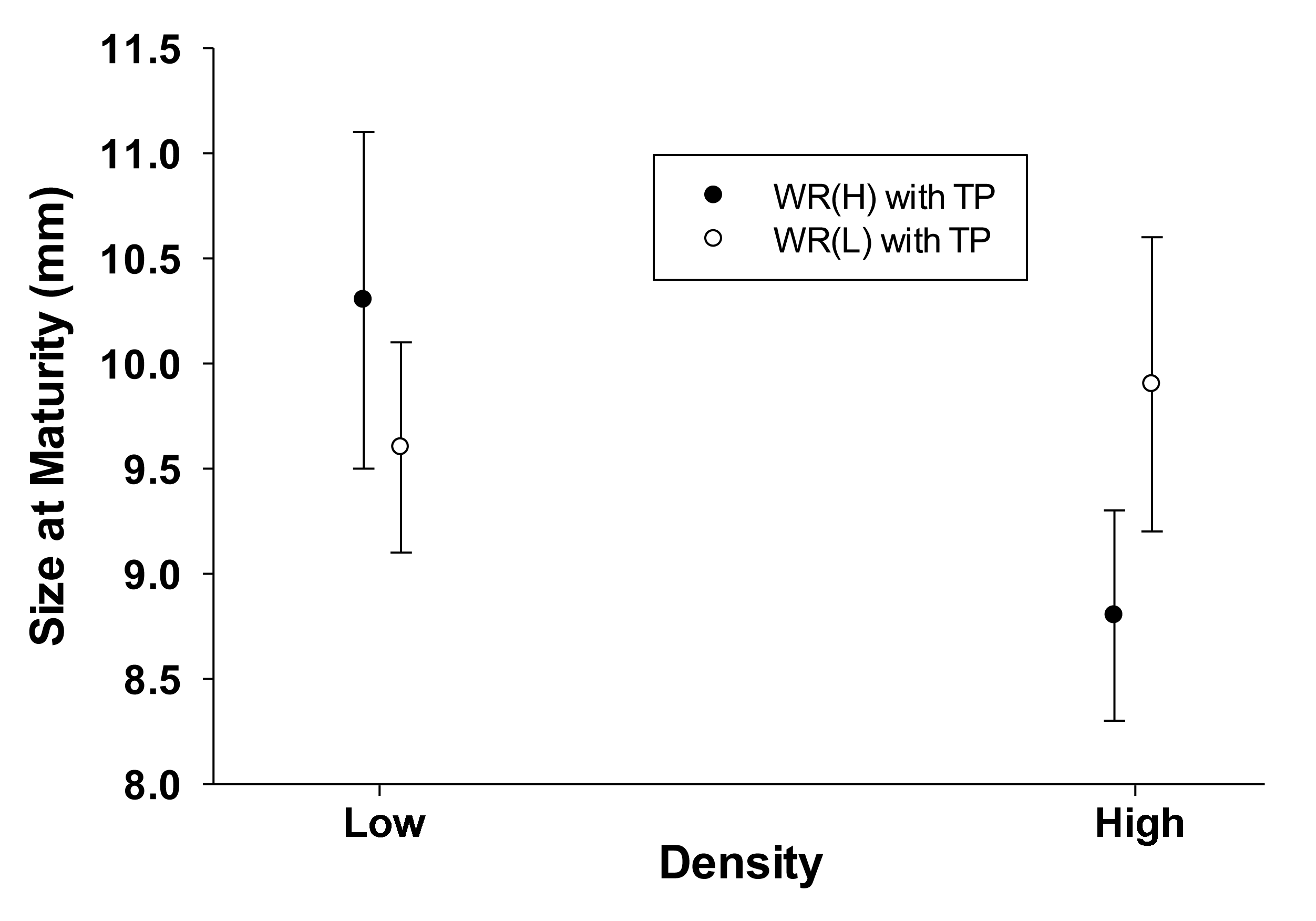

Supplement: Supplementary file 2 [file ece30003-0948-SD2.doc]
