# Supplementary material for: The adaptive significance of population differentiation in offspring size of the least killifish, Heterandria formosa
Source: Ecol Evol. 2013 Mar 5;3(4):948–60. doi: 10.1002/ece3.509 (PMC3631406; doi:10.1002/ece3.509)
Supplement: Supplementary file 3 [file ece30003-0948-SD3.doc]

**Supplementary Material**

**Table S1.** The top four rows re-express the information in Table 1. The top three rows indicate the identity and density of fish in that treatment level (Population MD = population and maternal density) and a fourth row that indicates the cell number used in Table 1 for that combination of TP (H), WR(H) or WR (L) fish. The remainder of the table is a set of contrasts among treatment levels that describe four two-way analyses of variance with different combinations of levels used in each analysis. The four analyses are designated by the Roman letters A-D. Within any single “analysis” (A, B, C, or D), there are three contrasts designated i, ii, and iii. The first two contrasts, i and ii, test for main effects and the third contrast, iii, tests for the interaction between two main effects. Analyses and hypotheses tested contrasts within each analysis are described in the text.

| **Population (MD)** |  | **Density Treatment** | | | | | | | | | | | | |
| --- | --- | --- | --- | --- | --- | --- | --- | --- | --- | --- | --- | --- | --- | --- |
| TP(H) |  | N | | 4N |  | 1/2N | | 0 |  | 2N | 0 |  | 1/2N | 2N |
| WR(H) |  | 0 | | 0 |  | 1/2N | | N |  | 2N | 4N |  | 0 | 0 |
| WR(L) |  | 0 | | 0 |  | 0 | | 0 |  | 0 | 0 |  | 1/2N | 2N |
| **Treatment code** |  | 1 | | 2 |  | 3 | | 4 |  | 5 | 6 |  | 7 | 8 |
| **ANALYSIS** |  | **Contrasts** | | | | | | | | | | | | |
| A | i | 1 | -1 | |  | 0 | 1 | |  | 0 | -1 |  | 0 | 0 |
|  | ii | 1 | 1 | |  | 0 | -1 | |  | 0 | -1 |  | 0 | 0 |
|  | iii | 1 | -1 | |  | 0 | -1 | |  | 0 | 1 |  | 0 | 0 |
|  |  |  |  | |  |  |  | |  |  |  |  |  |  |
| B | i | 1 | 1 | |  | -1 | 0 | |  | -1 | 0 |  | 0 | 0 |
|  | ii | 1 | -1 | |  | 1 | 0 | |  | -1 | 0 |  | 0 | 0 |
|  | iii | 1 | -1 | |  | -1 | 0 | |  | 1 | 0 |  | 0 | 0 |
|  |  |  |  | |  |  |  | |  |  |  |  |  |  |
| C | i | 0 | 0 | |  | -1 | 1 | |  | -1 | 1 |  | 0 | 0 |
|  | ii | 0 | 0 | |  | 1 | 1 | |  | -1 | -1 |  | 0 | 0 |
|  | iii | 0 | 0 | |  | -1 | 1 | |  | 1 | -1 |  | 0 | 0 |
|  |  |  |  | |  |  |  | |  |  |  |  |  |  |
| D | i | 0 | 0 | |  | 1 | 0 | |  | 1 | 0 |  | -1 | -1 |
|  | ii | 0 | 0 | |  | 1 | 0 | |  | -1 | 0 |  | 1 | -1 |
|  | iii | 0 | 0 | |  | 1 | 0 | |  | -1 | 0 |  | -1 | 1 |
